# Effects of NRF2 polymorphisms on safety and efficacy of bardoxolone methyl: subanalysis of TSUBAKI study

**DOI:** 10.1007/s10157-023-02427-w

**Published:** 2023-11-14

**Authors:** Kazuaki Ikejiri, Takafumi Suzuki, Satsuki Muto, Hirotaka Takama, Kengo Yamawaki, Tatsuya Miyazawa, Itaru Urakawa, Yuichi Aoki, Akihito Otsuki, Fumiki Katsuoka, Kengo Kinoshita, Masaomi Nangaku, Tadao Akizawa, Masayuki Yamamoto

**Affiliations:** 1 Research and Development Division, Kyowa Kirin Co., Ltd, 1-9-2 Otemachi, Chiyoda-ku, Tokyo, 100-0004 Japan; 2 Department of Medical Biochemistry, Tohoku University Graduate School of Medicine, 2-1 Seiryo-machi, Aoba-ku, Sendai, Miyagi 980-8575 Japan; 3 Tohoku Medical Megabank Organization, Tohoku University, 2-1 Seiryo-machi Aoba-ku, Sendai, Miyagi 980-8573 Japan; 4 The Advanced Research Center for Innovations in Next-Generation Medicine (INGEM), Tohoku University, 2-1 Seiryo-machi, Aoba-ku, Sendai, Miyagi 980-8573 Japan; 5 Division of Nephrology and Endocrinology, The University of Tokyo School of Medicine, 7-3-1 Hongo, Bunkyo-ku, Tokyo, 113-0033 Japan; 6 Division of Nephrology, Showa University School of Medicine, 1-5-8 Hatanodai, Shinagawa-ku, Tokyo, 142-8555 Japan

**Keywords:** Bardoxolone methyl, Chronic kidney disease, Diabetic kidney disease, Glomerular filtration rate, Nuclear factor erythroid 2-related factor, Single nucleotide polymorphisms

## Abstract

**Background:**

In the TSUBAKI study, bardoxolone methyl significantly increased measured and estimated glomerular filtration rates (GFR) in patients with multiple forms of chronic kidney disease (CKD), including Japanese patients with type 2 diabetes and stage 3–4 CKD. Since bardoxolone methyl targets the nuclear factor erythroid 2–related factor 2 pathway, this exploratory analysis of the TSUBAKI study investigated the impact of the regulatory single nucleotide polymorphism, rs6721961, on the effects of bardoxolone methyl.

**Methods:**

Japanese patients aged 20–79 years with type 2 diabetes and stage 3–4 CKD were randomized to bardoxolone methyl 5–15 mg/day (titrated as tolerated) or placebo for 16 weeks. Genotype frequency, clinical characteristics, renal function, and adverse events were primarily assessed.

**Results:**

Of 104 patients (bardoxolone methyl *n* = 55, placebo *n* = 49); 57% were genotype C/C, 32% C/A and 12% A/A. The frequency of the A/A genotype was higher among patients with diabetic kidney disease than in the general Japanese population (~ 5%). Measured and estimated GFRs increased from baseline in all genotypes receiving bardoxolone methyl. There were no significant differences between genotypes for safety parameters, including blood pressure, bodyweight, and levels of B-type natriuretic peptide, or in the type and frequency of adverse events, suggesting that the efficacy and safety of bardoxolone methyl are unaffected by the rs6721961 polymorphism-617 (C﻿→A) genotype.

**Conclusions:**

Our approach of combining genome analysis with clinical trials for an investigational drug provides important and useful clues for exploring the efficacy and safety of the drug.

**Trial registration:**

ClinicalTrials.gov; NCT02316821.

**Supplementary Information:**

The online version contains supplementary material available at 10.1007/s10157-023-02427-w.

## Introduction

Nuclear factor erythroid 2-related factor (NRF2) is the key effector molecule regulating the expression of antioxidant enzymes [1]. The action of NRF2 is negatively regulated by a thiol-rich protein called Kelch-like ECH-associated protein 1 (KEAP1) [2]. Animals without the NRF2 gene are incapable of upregulating antioxidant enzyme levels in response to oxidative stress [1].

There are a number of single nucleotide polymorphisms (SNPs) of the *NRF2* gene, but the most clinically relevant appear to be those in the upstream promoter region, specifically the rs35652124 (A → G) and rs6721961 (rSNP-617 C → A) polymorphisms [3–5]. Approximately 50–55% of healthy Japanese individuals carry the C/C genotype for rSNP-617, 35–40% carry the C/A genotype, and ~ 5% the A/A genotype [3]. Reportedly, the rSNP-617 C → A polymorphism significantly decreases *NRF2* mRNA expression [3].

Oxidative stress is a key pathogenic pathway in chronic kidney disease (CKD) [6], and research in nephrectomized animals indicates that the KEAP1/NRF2 pathway is impaired in CKD, with marked elevation of KEAP1 levels and a decline in NRF2 activity [7]. Bardoxolone methyl (RTA 402) is an oleanane triterpenoid that potently induces the phase 2 antioxidant response by inhibiting KEAP1 and activating NRF2 [8–10].

In several clinical trials, treatment with bardoxolone methyl significantly increased the estimated glomerular filtration rate (eGFR) in patients with type 2 diabetes and CKD [11–13], but the phase 3 BEACON (Bardoxolone methyl EvAluation in patients with Chronic kidney disease and type 2 diabetes mellitus: the Occurrence of reNal events) study was terminated early because of an increased risk of heart failure, which was likely caused by fluid overload in patients with stage 4 CKD [11].

TSUBAKI (Phase 2 Study of Bardoxolone Methyl in patients with Chronic Kidney Disease and Type 2 Diabetes Mellitus), the first study in patients with CKD after BEACON termination, was undertaken to determine the efficacy and safety of bardoxolone methyl in Japanese patients with type 2 diabetes and stage 3–4 CKD but without risk factors for heart failure [14]. None of the participants in the TSUBAKI study developed heart failure, and there was a significant improvement in GFR measured by inulin clearance compared with placebo [14]. There were also increases in the albumin:creatinine ratio (ACR) and liver enzyme levels in the bardoxolone methyl group compared with the placebo group in TSUBAKI [14], which is consistent with findings in the BEAM and BEACON studies [11, 13].

There is a possibility that SNPs of NRF2 affect renal function and/or the effects of bardoxolone methyl, which are mediated by NRF2 activity [15]. If so, NRF2 genotype may help to predict patients who may benefit from bardoxolone methyl or patients who are at increased risk of adverse events (AEs). Therefore, we undertook an exploratory analysis of the TSUBAKI study to investigate the impact of rSNP-617 genotypes (C/C, C/A, or A/A) on patient demographics and clinical characteristics, GFR and safety parameters in Japanese participants of that study. We also compared the characteristics of the TSUBAKI patients in each genotype subgroup with individuals with the same rSNP-617 genotypes from the Tohoku Medical Megabank Organization (ToMMo).

## Materials and methods

The methods of the TSUBAKI study (www.clinicaltrials.gov; NCT02316821) have been described in detail previously [14]. Briefly, TSUBAKI was a randomized, placebo-controlled comparative study conducted at 36 hospitals in Japan between December 2014 and September 2017. The study included Japanese patients aged 20–79 years with type 2 diabetes and stage 3–4 CKD, who were receiving treatment with angiotensin-converting enzyme inhibitors and/or angiotensin receptor blockers. Patients were excluded if they had a baseline B-type natriuretic peptide (BNP) level > 200 pg/ml or a significant cardiovascular history.

Patients were randomized to receive bardoxolone methyl or placebo for 16 weeks in two cohorts; in the stage 3 cohort, patients with stage 3 CKD (eGFR ≥ 30 and < 60 ml/min/1.73 m^2^) and ACR < 300 mg/g were randomized in a 1:1 ratio to bardoxolone methyl or placebo, and in the stage 4 cohort, patients with stage 4 CKD (eGFR ≥ 15 and < 30 ml/min/1.73 m^2^) and ACR < 2000 mg/g were randomized to these groups in a 2:1 ratio.

Bardoxolone methyl was administered orally once a day, starting at a dose of 5 mg/day and titrated as tolerated to 10 mg/day at week 4 and 15 mg/day at week 8. Patients were assessed weekly during active treatment, and then at 4-weeks intervals for 12 weeks after treatment completion. Assessment included vital signs (blood pressure [BP] and bodyweight), cardiac biomarkers (BNP, troponin T levels), renal function parameters (GFR, eGFR), and safety parameters (ACR, liver function tests, creatine kinase [CK] levels).

The primary efficacy endpoint was change in GFR from baseline to week 16 in the per-protocol stage 3 population; GFR was calculated using the inulin clearance method [16], and was measured at baseline and week 16 under controlled conditions during a 1–2 days hospitalization. The secondary endpoint was eGFR, estimated using the equation outlined by Matsuo and colleagues for Japanese individuals [16]. An exploratory endpoint was the eGFR in the full analysis set (FAS), considering both stage 3 and 4 cohorts.

At the beginning of the TSUBAKI study, the research plan for genome analysis was not fully elucidated, and therefore, it was stipulated in the study protocol that genome analysis would be performed as an exploratory investigation of individual differences in response to bardoxolone methyl and its relationship to DNA mutations such as polymorphisms. Approval for the study protocol and its amendments were obtained from the institutional review board at each study site and all work was carried out in accordance with the Declaration of Helsinki. The informed consent form (ICF) for the storage and use of blood samples for genome analysis was separate from the ICF for the TSUBAKI study participation. Whole blood samples for genome analysis were collected at baseline only from patients who provided written informed consent for both study participation and genome analysis. The genotyping substudy plan was established at the time of SNP analysis and approved by the ethics committee of Kyowa Kirin Co., Ltd. and ToMMo. Patients were also given the opportunity to exclude themselves from the substudy via an opt-out approach at study initiation. Genotype analysis of NRF2 rSNP-617 was conducted on the whole blood samples of patients who received at least one dose of bardoxolone methyl or placebo using TaqMan polymerase chain reaction (PCR) (study number M18-0090–01).

Separate control data were provided by the population of patients participating in the ToMMo study [17, 18], which was a cohort study conducted in approximately 8380 individuals between April 2013 and May 2020. Genotype information on NRF2 rSNP-617, patient background information (age, sex), and parameters of renal function (serum creatinine, eGFR, ACR), hepatic function (aspartate aminotransferase [AST], alanine aminotransferase [ALT], total bilirubin, and alkaline phosphatase [ALP]) and cardiac function (BNP levels) collected in the cohort study were used as controls in the current analysis.

### Statistical analysis

In the TSUBAKI study, the safety analysis set (SAF) included those patients who received at least one dose of bardoxolone methyl or placebo, and the full analysis set (FAS) included those patients who received at least one dose of bardoxolone methyl or placebo and had at least one post-baseline measurement of eGFR, while the per-protocol set (PPS) included those patients who received at least one dose of bardoxolone methyl or placebo and had available GFR data at week 16 [14]. This genome analysis included data from patients in each analysis set of TSUBAKI for whom genotype data were available. Consequently, the SAF for genome analysis was termed as SAFs, the FAS as FASs, and the PPS as PPSs to differentiate the patient population in this analysis from the analysis set of TSUBAKI cohort. Patients eligible for the PPSs also had no protocol violations, compliance of ≥ 80% or received study drug for ≥ 4 days in the last week of treatment, and a reliable measurement of inulin clearance at week 16.

In each treatment and genotype (C/C, C/A or A/A) group, parameters were calculated using the number of patients and percentage for categorical variables and the mean, standard deviation (SD), minimum, maximum, quartile 1 (25th percentile), median, quartile 3 (75th percentile), and 95% confidence intervals (95% CI) (t distribution) for continuous variables. No imputation was made for missing data.

The changes in GFR and eGFR assessed at week 16 were the PPS and FAS, respectively, using an analysis of covariance (ANCOVA) with treatment, genotype, and the interaction term between treatment and genotype as fixed effects, and baseline GFR/eGFR and log-transformed baseline ACR, as covariates. The least-squares mean (LSM) and 95% CI based on t-distribution for the change in each group was calculated.

Mixed-model for repeated measures (MMRM) were used to assess the changes in GFR and eGFR at each week, including intercept, treatment group, time, genotype, treatment group × time, and treatment group × genotype as fixed effects; and baseline GFR/eGFR and baseline ACR in logarithmic conversion as covariates. LSM and 95% CI were calculated for each treatment group and time combination, and the LSM difference between the two groups and 95% CI based on t-distribution were calculated for each week. SAS version 9.4 (or later) for Windows was used for the statistical analysis.

## Results

### Patient disposition

**Fig. 1:**
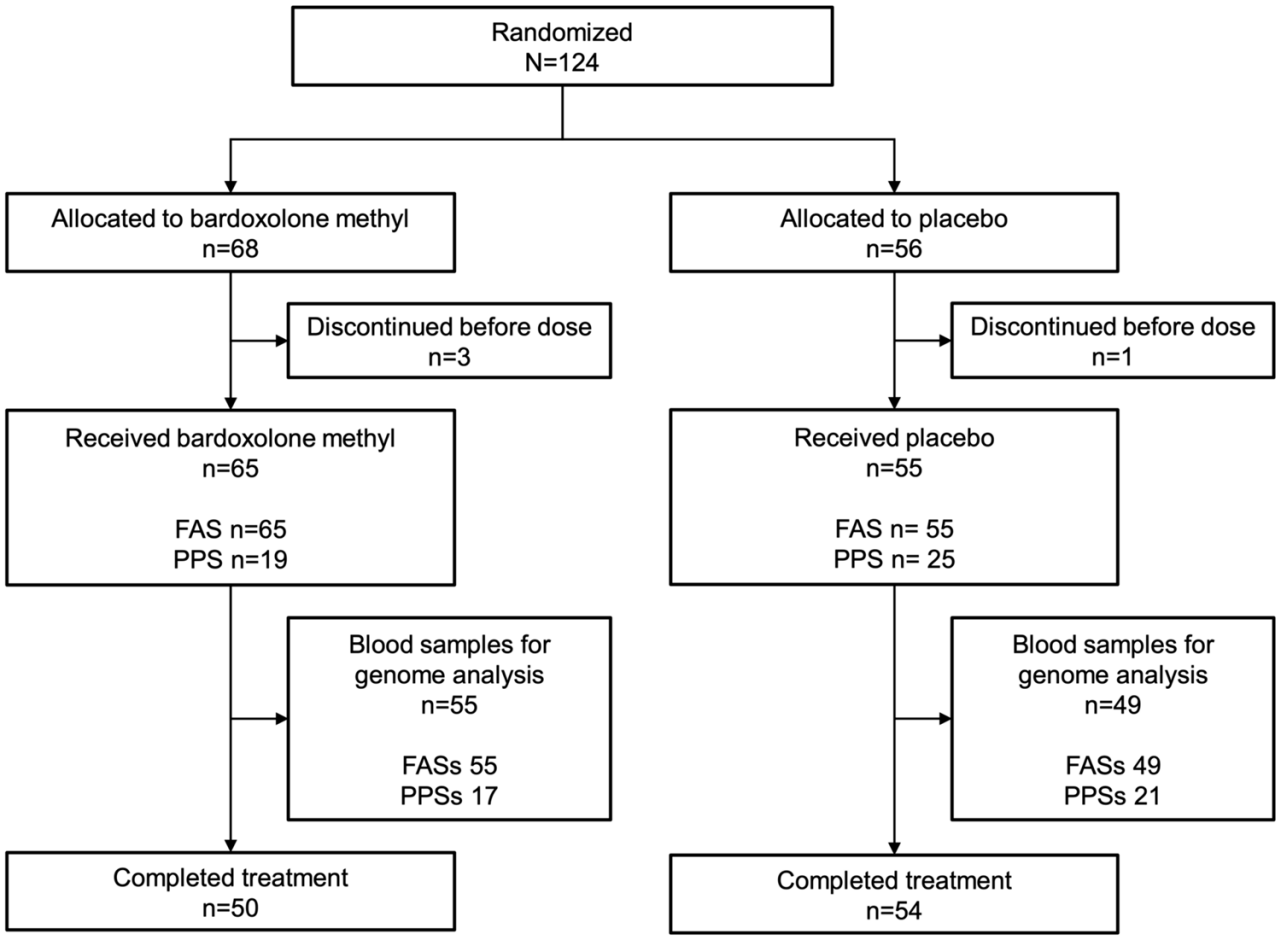
Patient disposition of all analysis sets included in the study. *FAS* full analysis set of the TSUBAKI study, *FASs* full analysis set of the genotype substudy, *PPS* per-protocol set of the TSUBAKI study, *PPSs* per-protocol set of the genotype substudy

Overall, genotype data were available for 104 patients. The FASs and SAFs consisted of 55 patients in the bardoxolone methyl group and 49 patients in the placebo group, and the PPSs consisted of 17 patients in the bardoxolone methyl group and 21 patients in the placebo group (Fig. 1).

In the 104 patients with genotype data and blood test results at week 16, 59 were genotype C/C (57%), 33 were genotype C/A (32%), and 12 were genotype A/A (12%). Demographic and clinical characteristics of the patients in each genotype group are shown in Table 1. The proportion of male patients was 78% (46/59), 61% (20/33), and 58% (7/12) in the C/C, C/A, and A/A genotype groups, respectively. Mean (SD) age ranged from 68 (8) to 69 (7) years across the genotype groups. Mean eGFR was 40.0 (12.7) ml/min/1.73 m^2^ in the genotype C/C group, 42.1 (12.0) ml/min/1.73 m^2^ in the C/A group, and 36.5 (11.1) ml/min/1.73 m^2^ in the A/A group.

**Table 1:** Patient demographics and clinical characteristics at baseline

	C/C genotype (*n* = 59)	C/A genotype (*n* = 33)	A/A genotype (*n* = 12)
Males, *n* (%)	46 (78)	20 (61)	7 (58)
Age, years	69 (7)	68 (8)	69 (5)
Weight, kg	68.6 (14.2)	66.3 (9.6)	65.0 (10.5)
BMI, kg/m^2^	25.5 (3.9)	25.5 (3.7)	25.4 (3.8)
CKD severity cohort in TSUBAKI, *n* (%)			
Stage 3	40 (68)	24 (73)	6 (50)
Stage 4	19 (32)	9 (27)	6 (50)
eGFR, ml/min/1.73 m^2^	40.0 (12.7)	42.1 (12.0)	36.5 (11.1)
GFR, ml/min/1.73 m^2^	49.3 (9.5)^a^	48.9 (7.8)^b^	52.0 (6.9)^c^
ACR, mg/g creatinine	292 (453)	391 (649)	462 (811)
B-type natriuretic peptide, pg/ml	34.0 (30.4)	32.6 (31.5)	47.3 (49.6)
Systolic blood pressure, mm Hg	130.8 (16.2)	132.2 (14.8)	120.5 (19.3)
Diastolic blood pressure, mm Hg	72.8 (9.6)	74.6 (10.0)	62.5 (8.2)
HbA1c, %	7.2 (0.9)	7.1 (0.8)	6.8 (0.8)

All data are mean (standard deviation) unless otherwise stated

*ACR* albumin:creatinine ratio, *BMI* body mass index, *CKD* chronic kidney disease, *(e)GFR* (estimated) glomerular filtration rate, *HbA1c* glycated hemoglobin, *TSUBAKI* phase 2 study of bardoxolone methyl in patients with chronic kidney disease and type 2 diabetes mellitus

^a^*n* = 31; ^b^*n* = 18; ^c^*n* = 4

Mean (SD) compliance rate overall was 99.55% (1.14) and did not differ between treatment or genotype groups.

### Comparison with the ToMMo cohort

The genotype data of 8,380 individuals from the ToMMo cohort were collected. Of these, 4,633 were genotype C/C (55%), 3,215 were genotype C/A (38%), and 532 were genotype A/A (6%). The proportion of individuals with the A/A genotype was higher in the TSUBAKI study than the ToMMo cohort.

The ToMMo cohort mainly consists of people in the general population, so the number of patients with diabetic kidney disease (DKD) in the cohort was small (*n* = 27). Of these 27 DKD patients, two were genotype A/A (7%).

### Change in renal function over time

GFR based on inulin clearance increased from baseline in all genotype groups who were receiving bardoxolone methyl. Genotype was not significantly associated with the change from baseline in GFR in the ANCOVA and the MMRM.

**Fig. 2:**
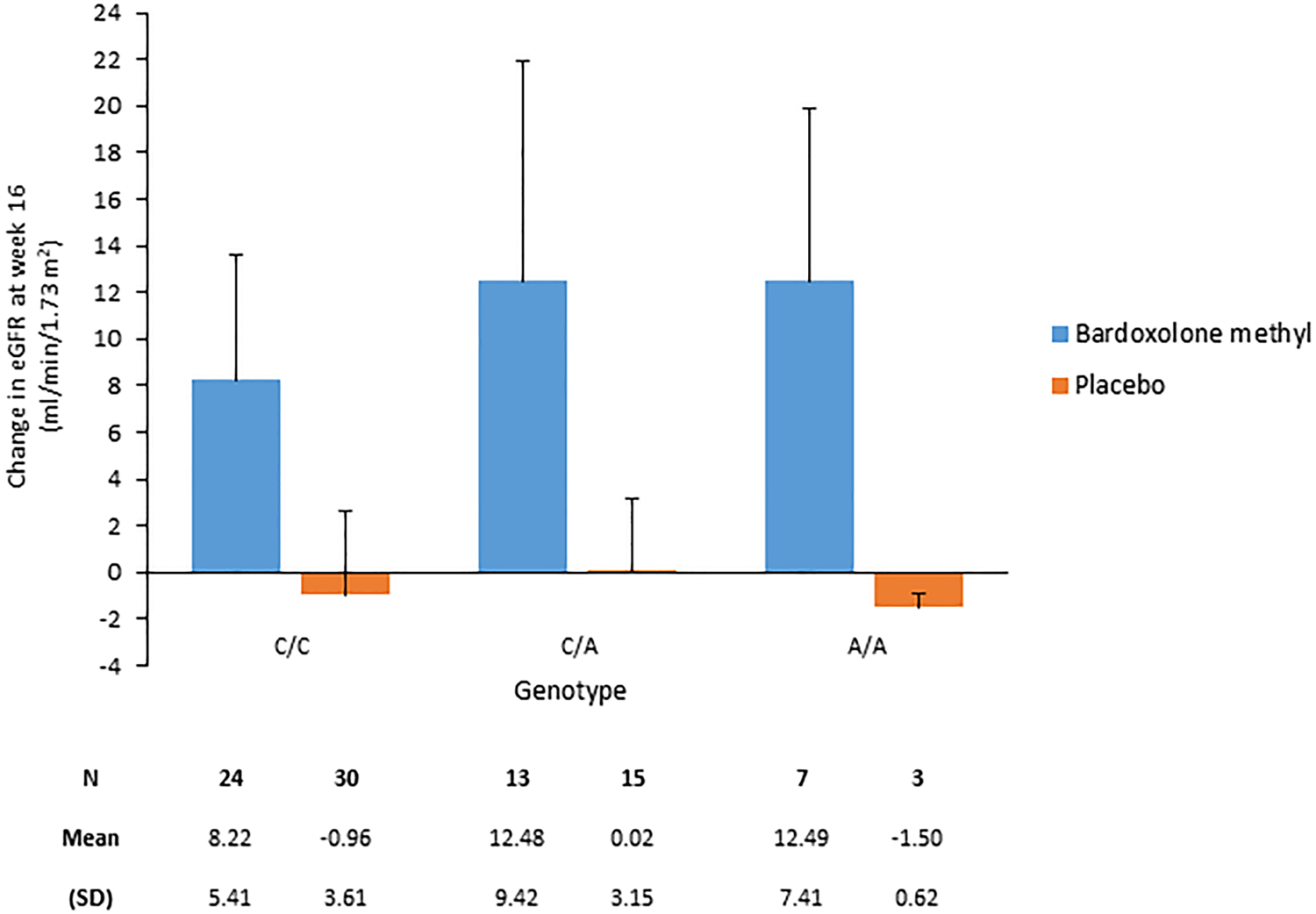
Change from baseline in estimated glomerular filtration rate (eGFR) at week 16 in FASs. *FASs* full analysis set of the genotype substudy

The mean (SD) eGFR increased from baseline at week 16 in patients receiving bardoxolone methyl, but showed no change or a slight reduction in patients receiving placebo (Fig. 2), and this was not significantly affected by genotype. The change in eGFR over time was similar in all genotype groups (Supplemental Fig. S1a and b; Online Resource 1). Four weeks after treatment completion (week 20), the least-squares mean change from baseline in eGFR in the bardoxolone methyl versus placebo group for each genotype was 2.69 (95% CI 0.90, 4.48) versus –1.52 (95% CI –3.04, 0.00) for genotype C/C, 3.18 (95% CI 0.31, 6.06) versus 0.55 (95% CI –1.55, 2.64) for genotype C/A, and 4.59 (95% CI 0.26, 8.92) versus –1.37 (95% CI –10.33, 7.59) for genotype A/A, respectively. Genotype was not significantly associated with the change from baseline in eGFR in the ANCOVA and the MMRM.

### Change in safety parameters

**Fig. 3:**
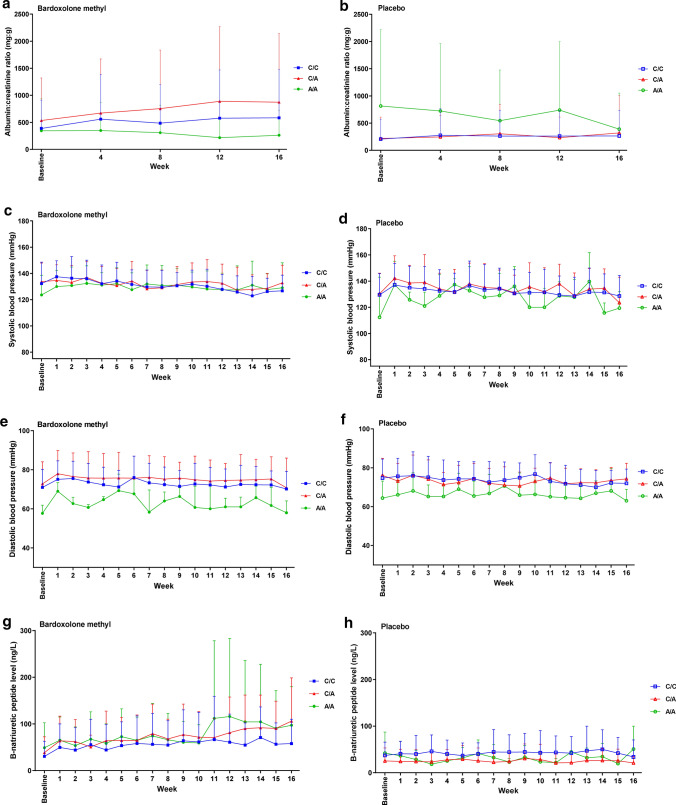

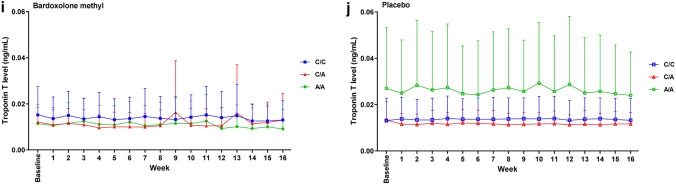
The time course of changes in (**a, b**) albumin: creatinine ratio (ACR), (**c, d**) systolic blood pressure (SBP), (**e, f**) diastolic blood pressure (DBP), and levels of (**g, h**) B-natriuretic peptide (BNP), and (**i, j**) troponin (T), in the two treatment groups of the FASs, stratified by genotype. *FASs* full analysis set of the genotype substudy

**Fig. 4:**
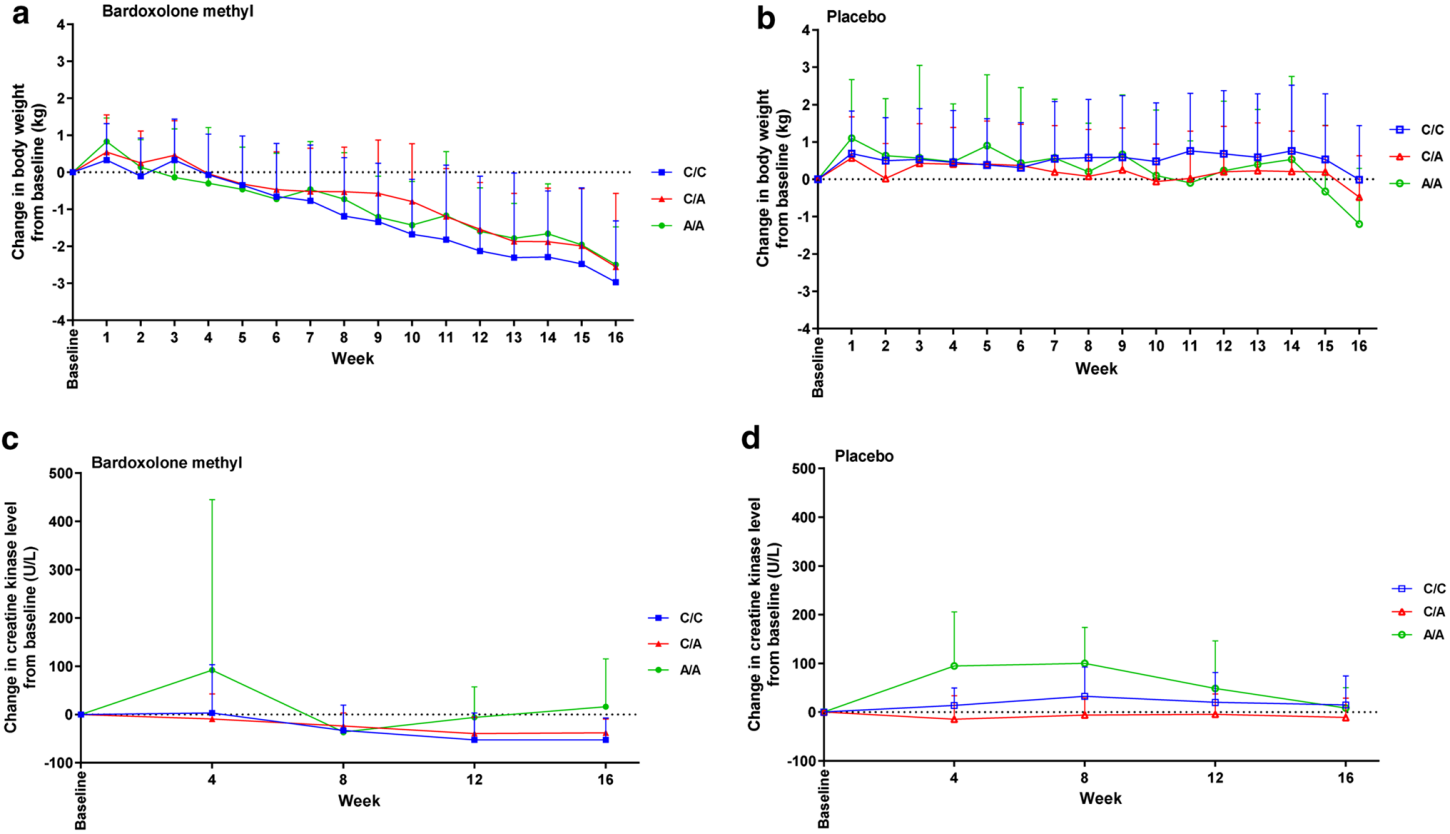
The change from baseline in (**a, b**) bodyweight, and (**c, d**) creatine kinase (CK) levels in the two treatment groups of the FASs, stratified by genotype. *FASs* full analysis set of the genotype substudy

There were no significant differences between genotype groups for any safety parameters. The change in ACR over time was not significantly different in the C/C, C/A or A/A genotype group (Fig. 3a and b). Systolic and diastolic BP did not change significantly over time (Fig. 3c–f). Mean BNP levels in patients receiving bardoxolone methyl tended to be higher in the C/A and A/A genotype groups than in the C/C group, but the SDs were wide and overlapped between groups, and there was no discernible pattern over the course of the study (Fig. 3g and 3h). Similarly, troponin T levels did not change significantly over time; these levels tended to be higher in patients with A/A genotype receiving placebo than in other groups, but there was considerable overlap in the SDs between groups (Fig. 3i and j). The change in body weight did not differ significantly between genotype groups (Fig. 4a and b) and CK levels did not change markedly during the course of the study, irrespective of treatment and genotype (Fig. 4c and d). Levels of AST and ALT tended to increase over time in the groups receiving bardoxolone methyl compared with placebo, but there were no significant differences between genotype subgroups (Supplemental Fig. S2a–d; Online Resource 1). Total bilirubin levels and levels of ALP did not differ significantly between treatment groups or genotype groups (Supplemental Fig. S2e–h; Online Resource 1).

### Adverse events

The type and frequency of AEs seen in the overall TSUBAKI analysis was generally similar when stratified by genotype (Table 2).

**Table 2:** Adverse events occurring in ≥ 15% of patients in any group

Adverse events, *n* (%)	Bardoxolone methyl			Placebo		
	C/C (*n* = 28)	C/A (*n* = 18)	A/A (*n* = 9)	C/C (*n* = 31)	C/A (*n* = 15)	A/A (*n* = 3)
Viral URTI	9 (32)	5 (28)	2 (22)	8 (26)	2 (13)	0
Constipation	6 (21)	2 (11)	0	2 (7)	1 (7)	0
Muscle spasms	0	3 (17)	2 (22)	0	1 (7)	0
Neck pain	0	0	0	0	0	1 (33)
Edema	0	0	0	1 (3)	0	1 (33)
ALT increased	13 (46)	5 (28)	3 (33)	1 (3)	0	0
AST increased	9 (32)	4 (22)	3 (33)	1 (3)	0	0
GGT increased	5 (18)	3 (17)	2 (22)	0	0	0

*ALT* alanine aminotransferase, *AST* aspartate aminotransferase, *GGT* γ-glutamyl transferase, *URTI* upper respiratory tract infection

## Discussion

This substudy of the TSUBAKI study is the first to investigate the impact of the rSNP-617 genotype on the efficacy and safety of bardoxolone methyl compared with placebo in patients with type 2 diabetes and CKD, and found that there was no significant relationship between these outcomes and genotype. In our cohort, 12% of patients had the A/A genotype, which is higher than the prevalence of this genotype in the ToMMo cohort (6%), and in previous reports among the general Japanese population, where the prevalence was 5% in a study from Tokyo [3], suggesting that the A/A genotype is present in a higher proportion of patients with DKD than in the general population.

A/A also appears to be the *NRF2* rSNP-617 genotype associated with an increased risk of clinical disease. A previous study showed that, compared with carriers of the C/A or C/C genotype, patients with cardiovascular disease who carry the A/A genotype are more likely to have multivessel disease [19]. Moreover, the A/A genotype is associated with an increased risk of cerebrovascular disease [20], and type 2 diabetes mellitus [21]. Wang and colleagues found that the A/A genotype of *NRF2* rSNP-617 was associated with significantly worse β-cell function and insulin sensitivity compared with the C/C genotype in a cohort of Chinese individuals composed of patients with newly diagnosed type 2 diabetes and healthy controls [21]. The rSNP-617 C → A polymorphism significantly decreases NRF2 mRNA expression, therefore, it was considered that the effect of bardoxolone methyl might be limited in the A/A genotype even if bardoxolone methyl inhibits KEAP1 from inactivating NRF2 [3]. However, no difference in the effect between genotypes was seen in this analysis. These results also suggest that bardoxolone methyl is a much more potent inducer than oxidative stress, such that the lower activity genotypes still elicit a sufficiently vigorous response. In addition, the safety profile did not differ between the genotypes, suggesting that treatment with bardoxolone methyl would not cause safety concerns in patients with a particular genotype at the initial stage of administration.

Data are currently limited on the impact of the NRF2 rSNP-617 genotype on renal disease development, but the pathogenesis of CKD is often multifactorial. As a result, it is likely that this polymorphism interacts pathogenetically with other comorbidities (such as diabetes and hypertension) and other genotypes, and that bardoxolone methyl may be acting on related factors other than NRF2 [22]. Among the DKD patients of the ToMMo cohort, the proportion of individuals with A/A genotype (7%) was similar to the proportion in the general population. The small proportion of A/A genotype in comparison with the TSUBAKI study was possibly due to the small DKD population in the ToMMo cohort, or may have resulted from differences in disease stage. The TSUBAKI population had a higher frequency of A/A genotype and more severe DKD compared with the ToMMo cohort, suggesting that compensatory mechanisms could be functioning to mitigate CKD progression in patients with mild DKD.

While our results found no association between the NRF2 rSNP-617 genotype and bardoxolone methyl efficacy and safety parameters, this does not preclude the possibility that such an effect exists. Our initial assessments of population size and study duration were based on the number of patients and time needed to demonstrate the primary endpoint in the overall study population and not in genotype subgroups. As a result, the A/A genotype subgroup included only 12 patients, so our study was probably underpowered to detect a difference between subgroups, resulting in large SDs and 95% CIs for some parameters.

Findings from the TSUBAKI study also suggest that combining genome analysis with clinical trials for an investigational drug might be helpful in exploring the efficacy and safety of the drug.

## Conclusions

Our analysis of TSUBAKI data stratified by genotype showed that bardoxolone methyl can be used in a wide range of patients regardless of genotype. The A/A genotype appears to occur at a higher frequency in patients with DKD than in the general population, suggesting that patients with the A/A genotype may benefit from renoprotective treatment. With regard to safety, no definitive conclusions can be drawn from the 16-week data in this analysis. Further studies in larger populations and with longer duration of follow-up are needed to establish a relationship between genotype and safety parameters during bardoxolone methyl treatment.

### Supplementary Information

Below is the link to the electronic supplementary material.

Supplementary file1 (DOCX 455 KB)

## Data Availability

Individual participant/patient data cannot be shared as the TSUBAKI study was conducted prior to January 1, 2019 and patient consent for secondary use of data by a third party was not obtained.
