# Supplementary material for: Effects of NRF2 polymorphisms on safety and efficacy of bardoxolone methyl: subanalysis of TSUBAKI study
Source: Clin Exp Nephrol. 2023 Nov 14;28(3):225–34. doi: 10.1007/s10157-023-02427-w (PMC10881689; doi:10.1007/s10157-023-02427-w)
Supplement: Supplementary file 1 — Supplementary file1 (DOCX 455 KB) [file 10157_2023_2427_MOESM1_ESM.docx]

**Effects of NRF2 polymorphisms on safety and efficacy of bardoxolone methyl: subanalysis of TSUBAKI study**

***Clinical and Experimental Nephrology***

**Online Resource**

**Authors:** Kazuaki Ikejiri^1^, Takafumi Suzuki^2,3^, Satsuki Muto^1^, Hirotaka Takama^1^, Kengo Yamawaki^1^, Tatsuya Miyazawa^1^, Itaru Urakawa^1^, Yuichi Aoki^3^, Akihito Otsuki^3^, Fumiki Katsuoka^3,4^, Kengo Kinoshita^3,4^, Masaomi Nangaku^5^, Tadao Akizawa^6^ and Masayuki Yamamoto^2,3,4*^

**Affiliations:** ^1^Research & Development Division, Kyowa Kirin Co., Ltd, Tokyo, Japan; ^2^Department of Medical Biochemistry, Tohoku University Graduate School of Medicine, Sendai, Japan; ^3^Tohoku Medical Megabank Organization, Tohoku University, Sendai, Japan; ^4^The Advanced Research Center for Innovations in Next-Generation Medicine (INGEM), Tohoku University, Sendai, Japan; ^5^Division of Nephrology and Endocrinology, The University of Tokyo School of Medicine, Tokyo, Japan; ^6^Division of Nephrology, Showa University School of Medicine, Tokyo, Japan

**Corresponding author:** Masayuki Yamamoto

**Email:** [masayuki.yamamoto.c7@tohoku.ac.jp](mailto:masayuki.yamamoto.c7@tohoku.ac.jp)

**Supplementary Fig. S1** Change in estimated glomerular filtration rate at each time point, stratified by genotype in the (**a**) bardoxolone methyl group and (**b**) placebo group


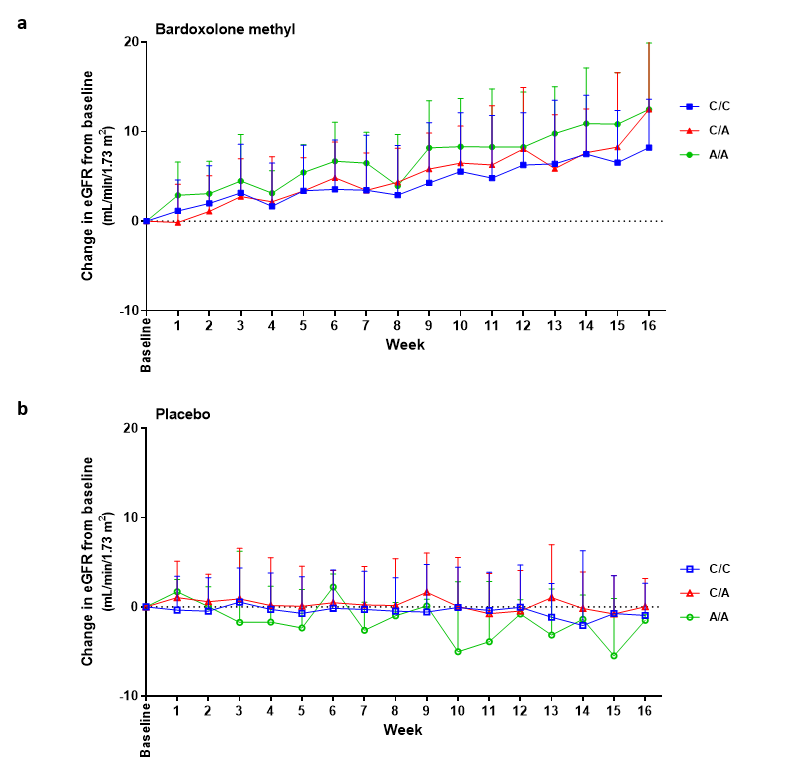


**Supplemental Fig. S2** Levels of (**a, b**) aspartate aminotransferase, (**c, d**) alanine aminotransferase, (**e, f**) total bilirubin, and (**g, h**) alkaline phosphatase at each time point, in the two treatment groups, stratified by genotype

**a**

**b**

**c**

**d**

**e**

**f**

**g**

**h**
